# Supplementary material for: Center-of-Mass iso-Energetic Collision-Induced Decomposition in Tandem Triple Quadrupole Mass Spectrometry
Source: Molecules. 2020 May 10;25(9):2250. doi: 10.3390/molecules25092250 (PMC7249026; doi:10.3390/molecules25092250)
Supplement: Supplementary file 1 [file molecules-25-02250-s001.pdf]

Supplementary materials to:

F. M. Rubino. *Center-of-Mass iso-energetic collision-induced decomposition in tandem triple quadrupole mass spectrometry.* Molecules 2020, 25, 2250

Correspondance to:
Federico M. Rubino. LaTMA Laboratory for Analytical Toxicology and Metabonomics, Department of Health Sciences, Università degli Studi di Milano at 'Ospedale San Paolo', v. A. di Rudinì 8, I-20142 MILANO (Italy), Lab.: +39-02-50323034 Federico.Rubino@unimi.it

List of Supplementary materials.

Table S1. Structures of the examined dabsyl-amino acids

Figure S2, S3. Measurement of representative CE_{max} and calculation of the scan line (DABS-AA)

Table S4. Structures of the examined nucleosides

Figure S5, S6. Measurement of representative CEmax of riboside transition MH+ @ BH+ and calculation of the scan line

Figure S7. Comparison of the generation efficiency curves of the BH+ fragment of protonated guanosine in Fragment Ion and in Neutral Loss spectra.

Figure S8. ESI source spectrum of a mixture of nucleosides.

Figure S9. Fragment ion spectrum of a protonated N6-subsituted adenosine (12)

Table S10. Experiments for the measurement of nucleosides.

Figure S11. Relative abundance of nucleosides in different conditions

Figure S12. Stability of signal in fast-scan *i*-CE Neutral Loss spectra

Figure S13. Comparison of CE_{lab} in a continuous and stepped scan of collision energy.

S1. Structures of the examined dabsyl-amino acids

	Compound name	Formula	M-H^{+}]$^{-}$	Structure
1	DABS-Gly	$C_{16}H_{18}N_4O_4S$	361	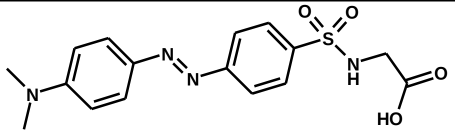
2	DABS-Val	$C_{19}H_{24}N_4O_4S$	403	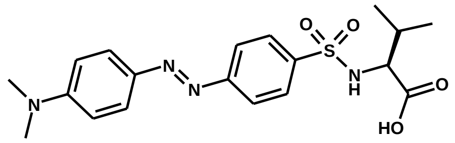
3	DABS-SMC	$C_{18}H_{22}N_4O_4S_2$	421	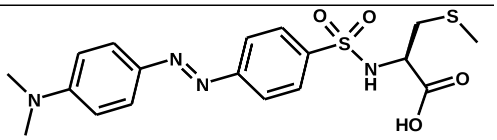
4	DABS-Met	$C_{19}H_{24}N_4O_4S_2$	435	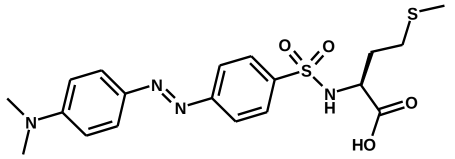
5	DABS-Asp	$C_{18}H_{20}N_4O_6S$	419	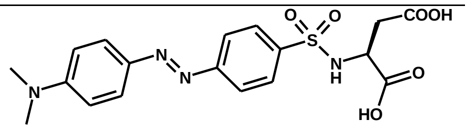
6	DABS-Glu	$C_{19}H_{22}N_4O_6S$	433	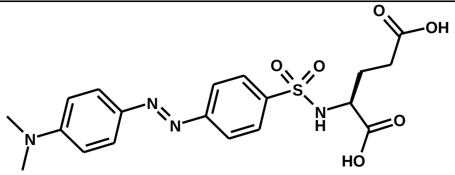
7	DABS-Trp	$C_{25}H_{25}N_5O_4S$	490	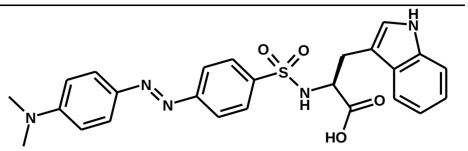

Figure S2, S3. Measurement of representative CE_{max} and calculation of the scan line (DABS-AA)

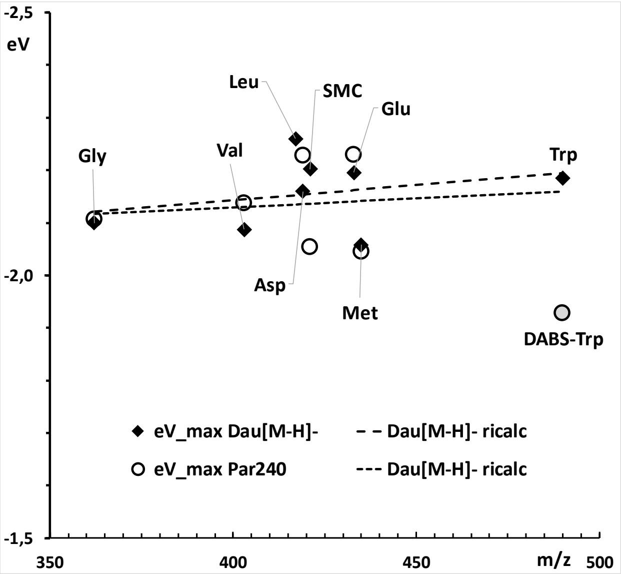

Figure S3.

Plot of calculated collision energy *vs.* precursor m/z for the maximum of the fragment formation efficiency of the characteristic transition of deprotonated DABS-AAs. Open diamonds are for curves derived from Fragment Ion spectra, open circles are for curves derived from Precursor ion spectra.

From the intercept of the best-fit lines

Y (eV) = ***1.91*** (eV) - 0,0006 * X (m/z) (R^2 = 0,0923)

Y (eV) = ***1.95*** (eV) - 0,0004 * X (m/z) (R^2 = 0,0237)

the mean value of the recalculated CE_{max} yields, for each series of measurements, the value employed to calculate the scan line of Figure S4 (right).

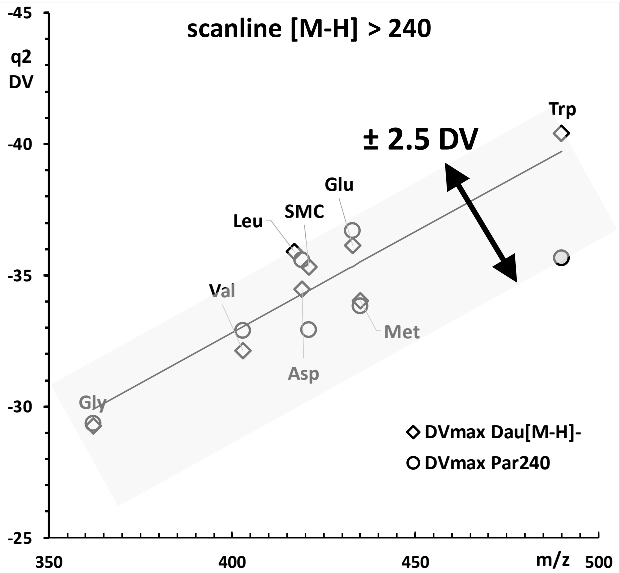

Figure S4.

Plot of measured collision voltage *vs.* precursor m/z for the maximum of the fragment formation efficiency of the characteristic transition of deprotonated DABS-AAs. Open diamonds are for curves derived from Fragment Ion spectra, open circles are for curves derived from Precursor ion spectra.

The gray band and its width, indicated by the double-pointed arrow, visualizes the width of the round-topped curve maxima (set at the value of 5 DV).

The best-fit line corresponds to the scan line calculated for CE_{max} = -2.15 eV.

Table S4. Structures of the examined nucleosides

	Compound name	Formula	MH+	Structure
1	2-deoxy-cytidine	$C_9H_{13}N_3O_4$	228	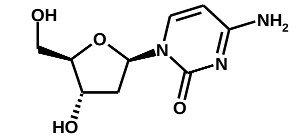
2	2-deoxy-thymidine	$C_{10}H_{13}N_5O_4$	243	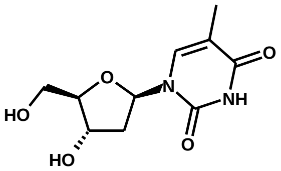
3	2-deoxy-adenosine	$C_{10}H_{13}N_5O_3$	252	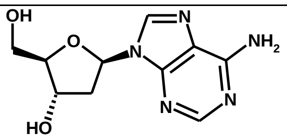
4	2-deoxy-guanosine	$C_{10}H_{13}N_5O_4$	268	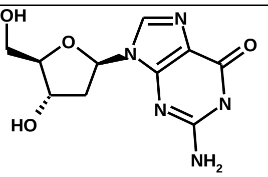
5	cytosine	$C_9H_{13}N_3O_5$	244	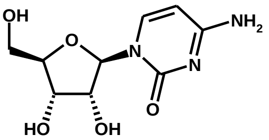
6	guanosine	$C_{10}H_{13}N_5O_5$	284	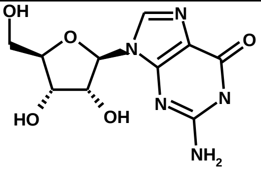
7	adenosine	$C_{10}H_{13}N_5O_4$	268	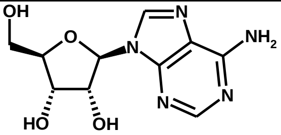
8	N6-isopentenyl-Adenosine	$C_{15}H_{21}N_5O_4$	336	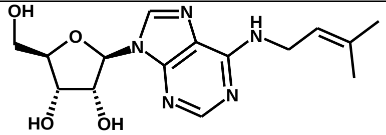
9	kinetin-riboside	$C_{15}H_{17}N_5O_5$	348	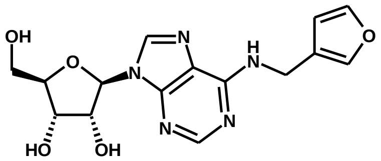
10	trans-zeatine-riboside	$C_{15}H_{21}N_5O_5$	352	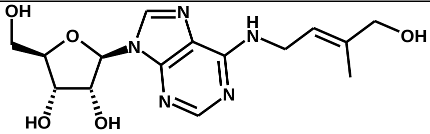
11	N6-Benzyl-Adenosine	$C_{17}H_{19}N_5O_4$	358	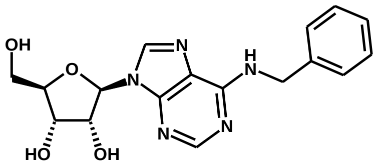

	Compound name	Formula	MH^+	Structure
12	N6-(4-OH-Benzyl)-Ade	$C_{17}H_{19}N_5O_5$	374	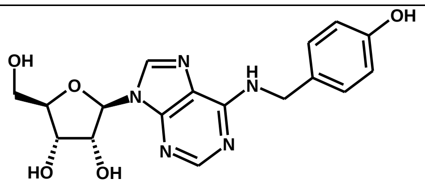
13	3,4-di-hydroxy-phenyl-ethyl-Adenosine	$C_{18}H_{21}N_5O_6$	404	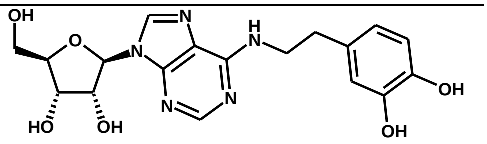

Figure S5, S6. Measurement of representative CE_{max} of riboside transition MH^+ @ BH^+ and calculation of the scan line

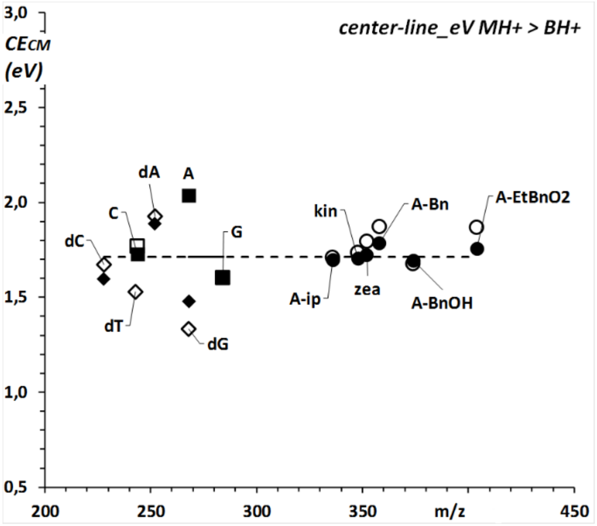

Figure S5.

Plot of calculated collision energy *vs.* precursor m/z for the maximum of the fragment formation efficiency of the characteristic transition of protonated ribo-nucleotides. Open diamonds are for curves derived from Fragment Ion spectra, open circles are for curves derived from Precursor ion spectra.

From the intercept of the best-fit lines

Y (eV) = ***1.57*** (eV) + 0,004 * X (m/z) (R^2 = 0,0518)

Y (eV) = ***1.49*** (eV) - 0,0008 * X (m/z) (R^2 = 0,0642)

the mean value of the recalculated CE_{max} yields, for each series of measurements, the value employed to calculate the scan line of Figure S4 (right).

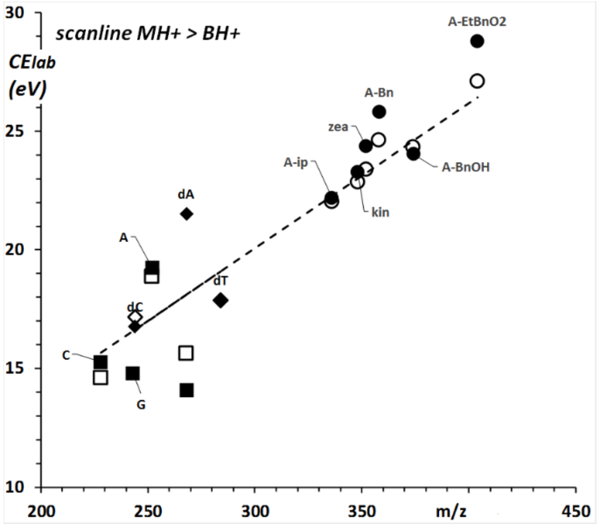

Figure S6.

Plot of measured collision voltage *vs.* precursor m/z for the maximum of the fragment formation efficiency of the characteristic transition of protonated ribo-nucleotides. Open diamonds are for curves derived from Fragment Ion spectra, open circles are for curves derived from Precursor ion spectra.

The best-fit line corresponds to the scan line calculated for CE_{max} = 1.71 eV.

Figure S7. Comparison of the generation efficiency curves of the BH+ fragment of protonated guanosine in Fragment Ion and in Neutral Loss spectra.

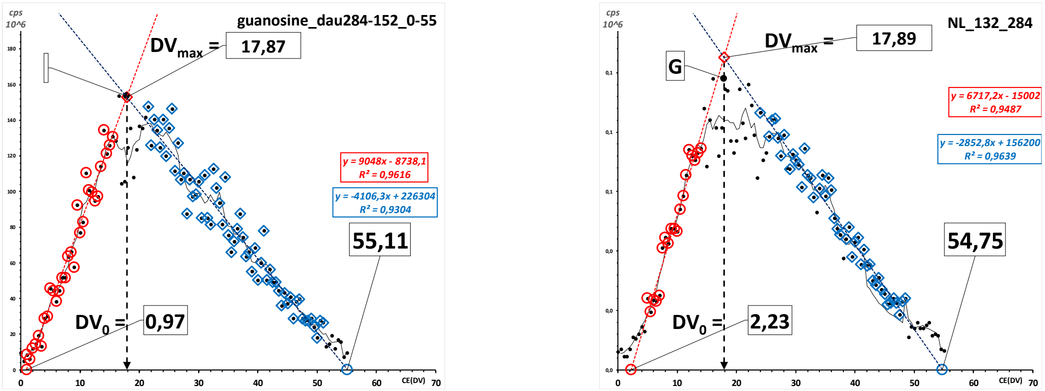

Figure S7. Comparison of the production efficiency curves of protonated guanine (m/z 152+ Th) recorded from protonated guanosine (m/z 284+ Th) in the Collision Energy Ramp (DV = 0.5 V) mode in Fragment Ion spectra (left) and in Neutral Loss of 132 Da spectra (right).

Figure S8. ESI source spectrum of a mixture of nucleosides.

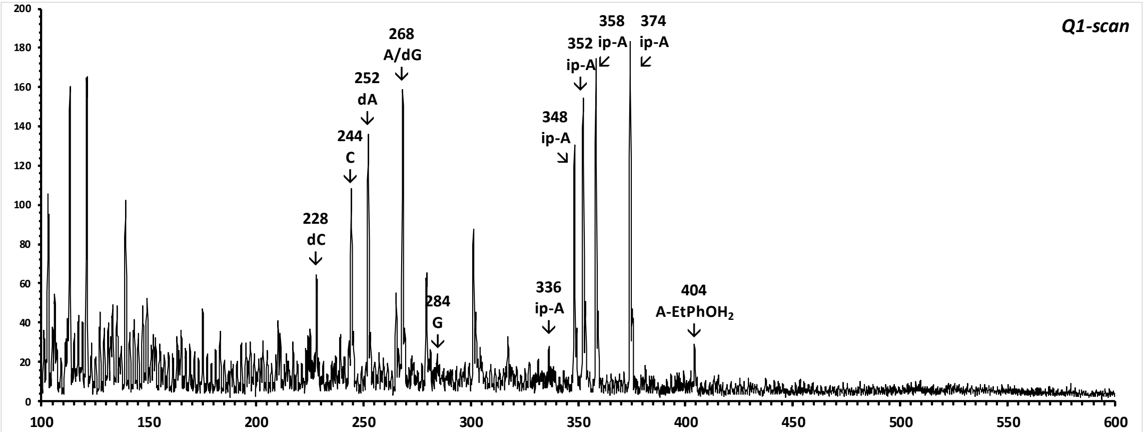

Figure S8. ESI spectrum of a mixture of nucleosides (1-13).

Figure S9. Fragment ion spectrum of a protonated N6-subsituted adenosine (12)

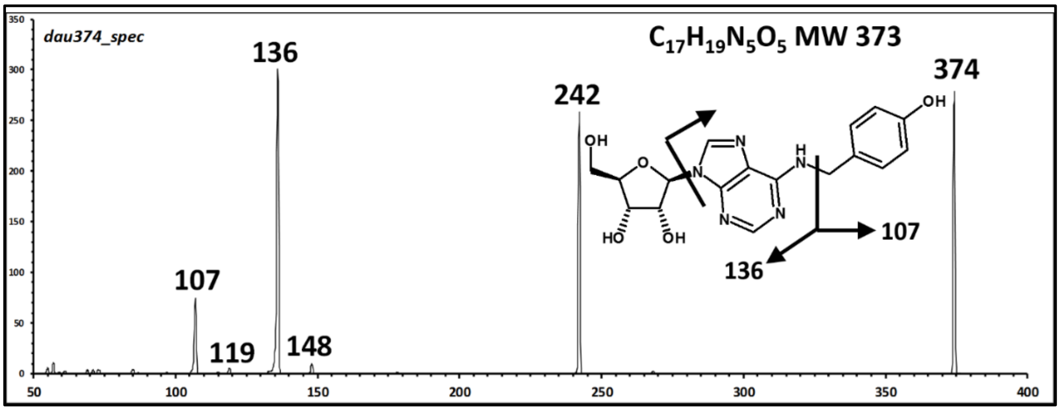

Figure S9. Integrated fragment ion spectrum of protonated N6-p-hydroxy-benzyl-adenosine (12). Collision energy 0-70 DV (0-4.88 eV).

Table S10. Experiments for the measurement of nucleosides.

Table S10. Setup of the experiments for the measurement of three RNA nucleosides and six modified adenine ribosides. The layout of the five rightmost columns corresponds to that of the instrument's data system.

Exp_ID	*Scan type*	*CE_{lab} (DV)*	*CE_{CM}(eV)*	*m/z START*	*m/z STOP*	*dwell (s)*	*CE_{lab} START*	*CE_{lab} STOP*
a	source scan			220	420	1.0		
b	NL132	Constant	1.58-0.88	220	420	1.0	14	14
c	NL132	Constant	2.26-1.25	220	420	1.0	20	20
d	NL132	Constant	2.94-1.63	220	420	1.0	26	26
e	NL132	ramp	1.65	220	420	1.0	14,6	26,4
f						0.5		

Figure S11. Relative abundance of nucleosides in different conditions

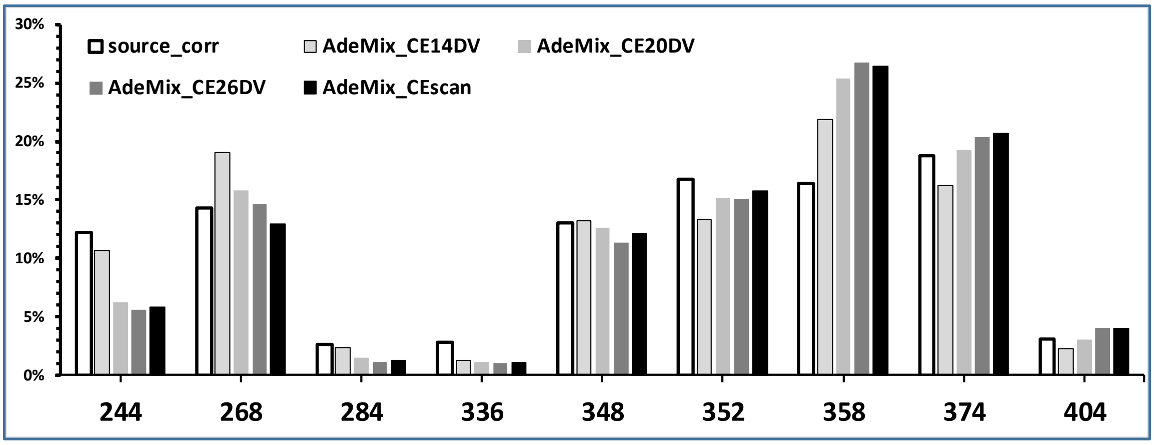

Figure S11. Comparison of the relative abundances of C, A, G, and six N6-substituted adenosine compounds in the source spectrum and in four different conditions of MS-MS detection (Table S11) at fixed laboratory CE_{lab} of 14, 20 and 26 eV(CE_{lab}) and with a synchronized scan of CE from 14.6 eV(CE_{lab}) (m/z 220) to 26.4 eV(CE_{lab}) (m/z 420).

Figure S12. Stability of signal in fast-scan *i*-CID Neutral Loss spectra

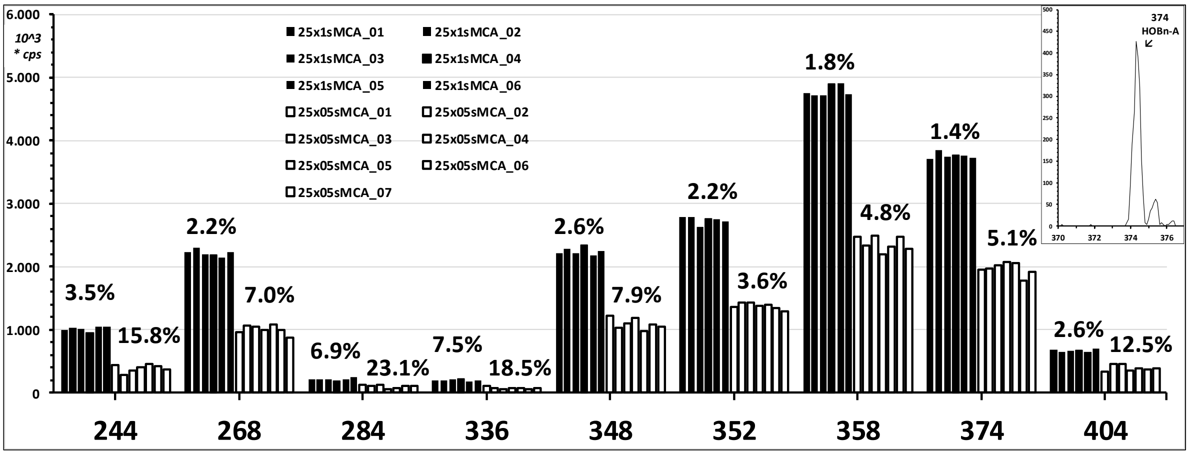

Figure S12. Stability of signals of ribosides **5-13** detected in a triple quadrupole with a synchronized (Neutral Loss of 132 Da) scan of Q1 and Q3, with simultaneous ramp of the collision voltage (q2-Q1). Scan speed is 1s (dark bars, condition *e* of Table S11) and at 0.5s (open bars, condition *f* of Table S11) over 200 Da(m/z). Insert is the profile of m/z 374 obtained in a single 0.5 s scan, showing no loss of mass resolution. Above each group of bars is the coefficient of variation (CV%) of the peak intensity.

Figure S13. Comparison of CE_{lab} in a continuous and stepped scan of collision energy.

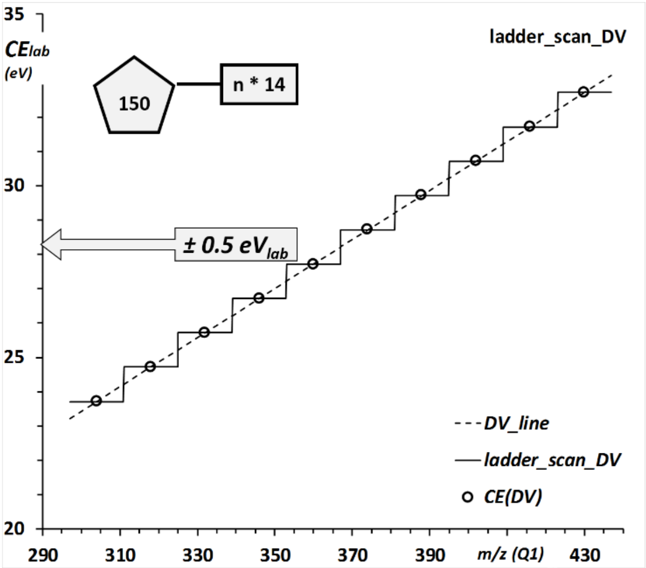

Figure S15_a. Comparison of CE(DV) in a continuous and stepped scan of collision energy.

A synchronized scan line of (q2-Q1) is calculated at CE_{cm} = 2.0 eV (dotted line).

A stepped *i*-CID Precursor Ion is calculated where Q1 scan is segmented in ten 14-u m/z ranges, each centered at the m/z corresponding to (150 + n*14), with a 14u width and CE_{lab}(eV) is calculated at CE_{cm} = 2.0 eV for the central m/z value (open circles).

The difference between the center value (open circles) and the value of the scan line at the lower and upper values of the scan range is ± 0.5 eV.
